# The extended nervous system: affect regulation, somatic and social change processes associated with mindful parenting

**DOI:** 10.1186/s40359-019-0313-0

**Published:** 2019-06-27

**Authors:** Kishani Townshend, Nerina Jane Caltabiano

**Affiliations:** 1 School of Medicine, The University of Adelaide, 55 King William Rd, North Adelaide, SA 5006 Australia; 2 The Cairns Institute, James Cook University, D3 McGregor Rd, Smithfield, QLD 4878 Australia; 3 James Cook University, Department of Psychology College of Healthcare Sciences, Division of Tropical Health & Medicine, McGregor Rd, Smithfield, QLD 4878 Australia

**Keywords:** Change mechanisms, Processes, Affect regulation, Somatic, Social, Mindful parenting

## Abstract

**Background:**

A theoretical model of mindful parenting has the potential to succinctly summarise its various change processes. The primary aim of this study was to investigate some of the change processes associated with mindful parenting, namely, the affect regulation, somatic and social change processes. A secondary aim was to verify whether clinical insights are consistent with the change processes identified in a systematic review of mindful parenting.

**Method:**

Interpretative Phenomenological Analysis (IPA) was used to analyse semi-structured interviews with four Australian clinicians delivering Mindful Parenting (MP) programs. The clinicians had extensive personal meditation practice. This qualitative study is part of a mixed methods study, which commenced with a quantitative systematic review.

**Results:**

Six higher-order themes identified as change processes included reflective functioning, attachment, cognitive, affective, somatic and social change processes.

**Conclusion:**

The anchor is a new theoretical model summarising the change processes associated with mindful parenting. The mother portrayed as the *extended nervous system* for the infant is a neologism that also has not been previously mentioned in the literature. Given the limitations with the small sample and potential bias with interpretation, the anchor is a starting point to developing a theoretical model of mindful parenting. Future research with larger sample sizes and objective measures is needed to confirm whether the anchor is a reasonable summary of the change processes.

Despite the escalating mental health expenditure, the rates of mental illness continue to rise in Australia. Expenditure on mental health services has recently surpassed $8.5 billion a year [1]. Yet, the system is still under pressure. Mindful parenting is a set of parenting skills broadly defined as the ability to pay attention to your child and your parenting in a particular way that is intentional, non-judgmental while being present-focused [2]. It is one of the many parenting programs currently being used as an early intervention tool. Understanding how Mindful Parenting (MP) programs are associated with changing parents’ behaviour is crucial in clarifying whether these programs are effective in reducing psychological distress.

Depression affects parenting, children’s health and psychological functioning [3]. The term *lost child* or *invisible child* is often used to describe the child of a parent with depression [4]. These children are considered *lost*, since much of the mental health treatments tend to focus on the parents and ignore the child. It is estimated that over a million children in Australia, approximately 23% of children under the age of 18 years, live with a parent with mental illness [5]. At least 15 million children are estimated to live in households with parents who have major or severe depression in the United States of America [6]. A cohort study of 86,957 parents in the United Kingdom found that by the time children reach 12 years of age, 39% of mothers and 21% of fathers had experienced depression as parents [7]. Children of parents with depression have been found to have a higher risk of developing affective illnesses, psychiatric problems [8] and medical problems [9] later in adulthood compared with children who did not have a parent with a mental illness. Although the association between maternal depression and children’s mental health is well established, further evidence is needed on how to assist these families.

## Attachment

Extensive research has consistently confirmed the quality of a child’s primary attachment relationships is the key determinant of a child’s socioemotional development [10–13]. Attachment is defined as “a strong disposition to seek proximity to and contact with a specific figure and to do so in certain situations, notably when frightened, tired or ill” [10]. The contemporary definition of attachment refers to the infant’s or young child’s emotional connection to an adult caregiver, an attachment figure as inferred from the child’s tendency to selectively seek that adult when experiencing distress [14]. The distinction between social engagement and attachment is that the child intentionally seeks the adult when distressed.

Four distinct patterns of attachment have been identified as secure, avoidant, ambivalent and disorganised [11, 15]. Secure attachment reflects a relationship in which the caregiver provides protection, *a haven of safety* for the infant’s emotional regulation when distressed [10] as well as support for the child’s exploration from a *secure base* [16]. Avoidant attachment is associated with caregiving responses that do not fully meet the child’s safe haven needs, with an overemphasis on encouraging exploration [11]. Ambivalent attachment is associated with unpredictable caregiver availability and/or inadequate support for secure base needs and reluctance to support autonomous exploration by the child [11]. Disorganised attachment occurs when the child experiences the caregiver as frightened or frightening [15, 17]. When infants expect the caregiver to provide safety, but instead experience danger, the infants were observed as being confused or frightened as regards their caregiver [15]. Psychopathology is strongly associated with disorganised attachment, leading to adverse emotional and behavioural outcomes for the children [18, 19]. Acknowledging these different patterns of attachment can assist parents in promoting secure attachment with their children.

Cortisol and oxytocin responses have been implicated in the quality of caregiving [20, 21]. While breastfeeding, secure mothers were observed to have strong decreases in cortisol, the stress hormone [22]. Oxytocin plays a crucial role in maternal bonding behaviour during pregnancy and postpartum period [23]. These maternal bonding behaviours include the gaze, *‘motherese’* vocalisations, positive affect, affectionate touch, attachment-related thoughts and frequent checking of the infant [23]. Lower levels of salivary oxytocin have also been found in not just the depressed mother, but her family, including the children and their father [21]. These children also had lower empathy and social engagement [21]. The implications of these findings are that insecure or traumatised mothers are more likely to have higher levels of cortisol and lower levels of oxytocin, which can be transferred to their infant.

The primary aim of this study was to examine the change processes associated with mindful parenting. The secondary aim was to verify whether clinical insights are consistent with the change processes identified in a systematic review of mindful parenting. Change processes that promote general mindfulness include *intention*, *attention* and *attitude* [24]. This paper uses the terms mechanisms and processes interchangeably. In fact, Shapiro, Carlson, Astin and Freedman [24] also use these terms interchangeably, as illustrated by the quotation, ‘*Intention, attention and attitude are not separate processes or stages’* (p. 375). Five core skills that facilitate mindful parenting are: (a) listening with full attention when interacting with their children; (b) non-judgmental acceptance of self and child; (c) emotional awareness of self and child; (d) self-regulation in the parenting relationship; and (e) compassion for self and child [25]. Change mechanisms that specifically promote mindful parenting have been identified as attachment, emotional awareness, intentionality, compassion and kindness [26]. A systematic review on mindful parenting summarised possible change mechanisms identified in literature as intention, attitude, attention, affect regulation and attachment [27, 28]. The substantive research question driving this study was, *what are the change processes associated with Mindful Parenting?*

## Methods

Whilst all qualitative methodologies allow for a degree of epistemological flexibility, Interpretative Phenomenological Analysis (IPA) was the most appropriate methodology to answer this study’s research question. IPA is a useful methodology for theory development, transferability and understanding processes operating within models [29]. Its theoretical roots in psychology lends itself to understanding the clinicians’ perspective or lived experience from a phenomenological sense. Experts in the field were interviewed for their insights from extensive meditation practice and wealth of experience observing how parents change through attending the Mindful Parenting (MP) programs. Smith and Osborn [30] recommended a sample size of three for students performing IPA for the first time. Following recommendations by Smith and Osborn [30], this study recruited a purposive sample of four clinicians delivering MP programs.

**Fig. 1:**
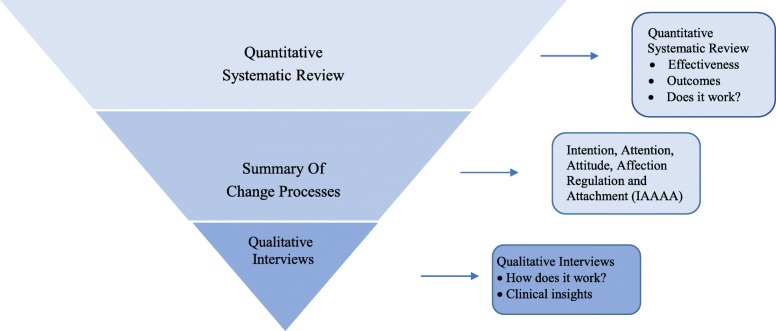
Mixed methods study design investigating the change processes associated with mindful parenting

Figure 1 illustrates the mixed methods research design, which led to this qualitative interview study. The first stage of this study was a systematic review that investigated the effectiveness of MP programs. The second stage summarised the numerous change processes identified in the systematic review into five categories, namely Intention, Attention, Attitude, Affection Regulation and Attachment (IAAAA). The third stage is this qualitative study, which aimed to verify whether the clinical insights on the change process associated with mindful parenting are consistent with those identified in the literature.

### Procedure

A purposive sample of four was used since MP programs are not widely used in Australia. It was difficult to recruit facilitators because few clinicians deliver this program in Australia. The clinicians were accredited by the peak training body for mindfulness teachers in Australasia, the Mindful Training in Australia and New Zealand (MTI ANZ). Only clinicians could be interviewed under ethics approval, not the parents. Ethics approval was granted by the Human Research Ethics Committee (HREC) at an Australian university (H-2017-080) and maternity hospital (HREC/16/WCHN/21) for a Low and Negligible (LNR) ethics application. Ethics approval was required from the maternity hospital to interview their clinicians. Since the research was part of a PhD project, ethics approval was also sought from the university to interview clinicians outside the hospital. Contact details of potential participants were accessed through the professional networks for mindfulness programs in Australia.

All four participants who were emailed by the first author agreed to be interviewed. The interview questions 1 to 10 outlined in Table 1 were emailed to the participants a week before the interview. Question 11 was not emailed to the participants prior to the interview to prevent influencing the participants’ responses. All participants signed the consent forms. The semi-structured interviews were conducted according to guidelines provided by Yin [31] and Smith, Flowers and Larkin [32]. The interviews occurred via Skype while the participants were in their homes or private office.

**Table 1:** A list of the interview questions

	Questions
1	How long have you been working as a mental health professional?
2	What does your role entail?
3	How did you become interested in Mindful Parenting?
4	What is Mindful Parenting?
5	What is the theoretical basis of Mindful Parenting?
6 a)	How is the course structured?
b)	How many hours of training do they attend each week?
c)	What is the course content?
d)	What is done in the classes? Is it a combination of information provision, self-reflection and group therapy?
f)	What aspects of the group dynamics promote insight/behaviour change?
7)	What are the crucial elements/the active ingredients of this program that promote behaviour change?
8)	What psychological processes do you think facilitate behaviour change?
9)	Share with us some examples of how it has changed your participants’ thinking, feelings, behaviour and parenting.
10 a)	Have you observed any examples of how it may have influenced the participants’ children?
b)	Have you noticed any differences in the birthing process, birth weight and on the child as they grow?
11)	Some of the change processes identified in the Mindful Parenting literature could be grouped under 5 headings: -
a)	Intention (Intentionality, Re-perceiving, Listening)
b)	Attitude (Non-judgmental acceptance, compassion)
c)	Attention (Attention to variability, attention regulation)
d)	Emotion (attunement, emotional awareness, affect regulation)
e)	Attachment (secure attachment)

An audio recorder was used to tape the interviews, which were later transcribed in full. The duration of each interview was approximately 60 min. All participants were asked the same questions to gain consistency with information gathering about their background, experience, role, program content, group dynamics and change processes.

### Participants

Four Australian, female clinicians delivering MP programs were interviewed once via Skype. The age of the participants ranged from 35 to 65 years. The clinicians were accredited by MTI ANZ. The clinicians maintained regular personal meditation practice, attendance at yearly retreats, regular peer support and supervision. Ideally, the researchers would also interview the parents. However, ethics approval was not granted to interview the parents. This paper used the pseudonyms Anna, Bella, Cara and Diana to protect the privacy of the participants. The participants lived in different Australian locations. Skype was used to interview the participants as it was the most cost-effective data collection strategy. Anna and Cara delivered a combination of the Mindfulness Based Stress Reduction (MBSR) and Circle of Security (COS) referred to as COS-*M. Bella* and Diana delivered the *Caring for Body and Mind in Pregnancy* (CBMP) program, which is an adaptation of Mindfulness Based Cognitive Therapy (MBCT) to the perinatal context. All clinicians had at least one child of their own, except for Cara. The participants were mental health clinicians and accredited mindfulness facilitators with extensive personal meditation practice of over two decades.

Anna was a psychotherapist with over 30 years of experience working as a psychotherapist, 13 years of experience delivering MBSR and 3 years of experience delivering COS. Her training was in Body-Oriented psychotherapy, Psychodynamic psychotherapy, Self-Psychology, Attachment Theory and trauma. Bella was a perinatal psychiatrist with over 20 years of experience treating parents presenting with a range of issues, including persistent difficulties with trauma, attachment, settling and emotional regulation. She had over 8 years of experience delivering MBCT and CBMP. She was experienced in early intervention from conception to postpartum infant mental health. Cara was a psychotherapist with 7 years of counselling experience and 3 years of delivering the COS-M program. She was an experienced meditator with over 20 years of experience living in Sri Lanka during the civil war. Diana holds a Doctor of Philosophy degree. Diana had 7 years of experience delivering the CBMP program as well as 16 years of experience counselling women presenting with depression, anxiety and perinatal mental health issues.

#### Program

Two distinct MP programs were delivered by the participants in this study. Bella and Diana delivered the CBMP, whereas Anna and Cara delivered COS-M. The similarities between the programs are that both entwined two divergent epistemologies, the Eastern contemplative practice with the Western Cognitive Therapy and Attachment Theory. CBMP is strongly based on MBCT, while COS-M is based on MBSR. Both programs were 2 hrs per week in duration for 8 weeks. A one-day retreat in Week 5 was included in both programs. The principles of MBSR and COS were utilised by both programs. This included attachment, *shark music,* relating to their child and MBSR techniques. Shark music refers to a video from the COS program that raises parents’ awareness about perception and fear. Both courses used MBSR techniques, such as the body scan, breathing space, observing thoughts, replacing fear with curiosity and sitting meditation. Similarly, both courses used the term *home-based practice* rather than *homework* for practice conducted at home. However, the required duration of home-based practice varied. COS-M encouraged 40 min of sitting meditation, whereas CBMP encouraged shorter periods until participants were able to sit for longer periods of 30 min. An emphasis by all clinicians was that parents were not *forced* to do *homework,* instead they were encouraged to *practice* at a consistent time each day that suited their schedule.

#### Data analysis

IPA was utilised to analyse the data in four stages as recommended by Smith and colleagues [30, 33]. During the first stage, the transcripts were read several times and organised into a table. The raw data were in the first column, the explanatory notes were in the second column and the themes in the third column. The first author read the transcript several times during the first stage, then made explanatory notes in the second column with quotations that appeared significant. With each reading, the researcher became more responsive, becoming more *wrapped up* in the data. During the second stage, the initial notes were transformed into themes in the third column by linking them to psychological constructs where possible. The preliminary themes were then further reduced to higher-order themes with subtheme clusters during the third stage of data analysis. The final product was a table with each higher-order theme, the related subthemes and a brief illustrative data extract for each theme [33]. To preserve the integrity of the participants’ voice, caution was exercised to ensure the researcher’s interpretations accurately reflected the participant’s own words. The second author conducted an independent audit and tracked the raw data to the final table. The writing process continued the data analysis by organising the interplay between the researcher’s interpretation and the participants’ words into an overarching *gestalt*. Table 2 illustrates how the data were analysed to maintain technical rigor.

**Table 2:** Example of how the transcripts were analysed to produce explanatory notes and then themes

	Transcript	Explanatory Notes	Themes
Diana	*So I think it’s really hard to know beforehand. All you can do is explain to people exactly what the class involves and you know let them know that some people have found that it isn’t helpful um. You know, that their anxiety can go sky high um, well, what we would tend to do if it happened in class, is get people* ***to stop doing that meditation*** *and get them to maybe* ***focus on something outside of their body*** *um like an* ***object or sound*** *or something like that* ***rather than focus on their breath or their body which is often the trauma holder***. (p.32)	Focus attention outside the body like an object or sound rather than their breathing or their body, which is often the trauma holder	Body
			Body as a trauma holder
Diana	*And always like recently we had someone who was um, you know persisting with the* ***body scan*** *with a very high trauma background. And um but really … what we got her doing is we got her to stop doing that but she found that the* ***mindful movement*** *um, didn’t aah, she didn’t get overwhelmed in that and actually it kind of* ***deescalated*** *things for her. So um you know you can work with them … I think you have to be* ***very careful*** *and you know respond to each individual. And I mean sometimes you know we might say no to someone joining the class because you know their vulnerability*. (p.32)	Mindful movement more helpful than body scan for participants with a trauma background	Body
		Role modelling self- care	
		Some participants are not allowed to join because of their vulnerability	
Diana	*Yeah so it varies … what some people report is that um they have noticed that if they are more* ***present*** *with the baby, say when breastfeeding something like that baby seems to be … you know the* ***feeding process seems to go perhaps better for the baby****. … … … she noticed that when she was sort of doing the* ***breathing space*** *or just a* ***mindful breathing*** *so she was not shallow breathing. And she noticed her* ***baby’s breathing came in rhythm with hers as well***. (p.45)	Being present, mindful breathing influenced the baby	Breath, body
Interviewer	*Tell me about the 3 min breathing space. What does it entail?*		
Bella	*Well that’s the … That’s the meditation short practice, that’s introduced in uh, class three. Uh and it’s a* ***3 min check in, I guess with your internal state****. So it, it’s asking the question,* ***what’s going on right now, in my thoughts, feelings and bodily sensation****. And it’s asking that question also with no judgment and with acceptance. Um, then this is a short, uh focus on the breaths of bringing the attention to breath again, um allowing the breath to open up to* ***how it feels in the body*** *and the third part of it. So this is generally 1 min each*.	Three minute breathing space likened to an hour glass	Attention, breath and body
	*The third part is* ***bringing your attention focus into the whole of the body with the breath****. So, they say it’s shaped like an* ***hour glass****, it starts wide with your attention, narrow down and then widens out again*. (p.10–11)		
Cara	*The training is in Melbourne and is called Somatic Experiencing and it’s a trauma resolution mode. Uh you know, trauma can be attachment, it can be car accidents, it can be emotional abuse, it could be physical abuse, sexual abuse. And it, it really is basically* ***using mindfulness in the body*** *to help regulate people so that those patterns that keep people acting over and over again in the same way, it just, it just unravels. It’s really beautiful. Very effective and direct*. (p.30)	Using mindfulness in the body to regulate unhelpful patterns.	Body

#### Reflexivity

Reflexivity is an important part of all qualitative research studies. To maintain the methodological rigor and reliability, the clinicians were given a copy of their transcripts to verify whether they agree with the content. The second author also conducted an independent audit to track the raw data to the final table. To the authors’ knowledge, the findings are reliable because the reiterative process checked whether the clinician’s raw data accurately reflected the researcher’s interpretation. The authors’ role and background also had the potential to influence data collection, data analysis, the way questions were asked, interpretation of results and how this was managed**.** The first author’s experience working as a psychologist with families from diverse cultures could have influenced both the data collection and analysis, particularly designing the interview questions on understanding how parents change. The second author’s extensive experience with psychological research and parenting influenced data collection and analysis to ensure methodological rigor. All attempts were made to minimise potential bias by being as transparent as possible and reflecting on the authors’ potential biases.

## Results

**Fig. 2:**
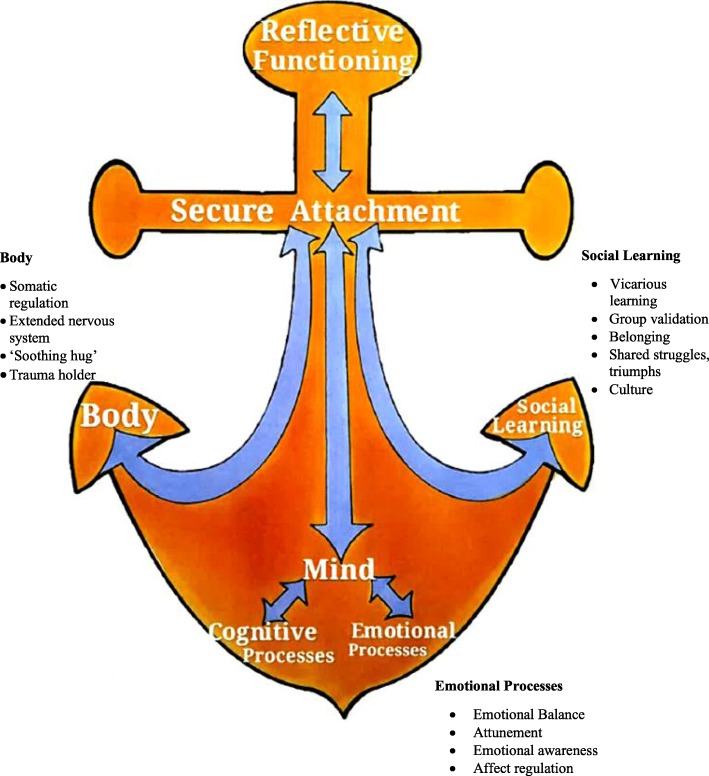
Anchor: A theoretical model of Mindful Parenting

Six higher-order themes emerged from the data analysis. Figure 2 summarises the themes identified in the transcripts. This paper focuses on how somatic, emotional and social learning processes facilitate mindful parenting.

### Somatic mechanisms

All the clinicians highlighted body awareness as a critical change process. The body scan is a frequently used mindfulness technique, used to increase breath awareness and identify stressors and feelings in the body. The importance of whole-body awareness is a recurring theme. Anna commenced her clinical training in body-oriented psychotherapy. Anna trained parents to read their child’s body language and posture. She trained parents to look at their child’s eyes to identify their child’s needs. Diana described how mindful breathing improves breastfeeding. Bella spoke about a mother with severe depression who was unable to take her medication for restless legs during pregnancy. This mother had experienced interrupted sleep and ongoing aggravation:

*She was… responding to the restless legs with a whole lot of judging thoughts about, I shouldn’t have this, and my father had it and I didn’t like my father… the thoughts went around in* [a] *...ruminating frustrating way.… as soon as she recognised that, that was the process, she had this aha moment and she was able to drop the judgment that having this unpleasant experience in her body. It became much, much easier for her to tolerate the actual physical experience… she was able to sleep better*.

Recognising the habits of the mind was a process the mind frequently engaged in, resulting in the reduction of the physical symptoms.

The association between trauma, neglect and the physiology of the developing brain emerges in all the interviews (Anna, Bella, Cara). Diana described the body as being the “*trauma holder.”* Likewise, Cara described how the “*body keeps score,”* mentioning Bessel van der Kolk’s book and Peter Levine’s work on *Somatic Experiencing.* Bella highlighted how memories of sexual assault often arise during childbirth. Cara illustrated the importance of a “*soothing hug”* and physical contact as being essential for healthy development. Neglect and the lack of social contact also impair healthy development. To highlight this, Cara provided the example of the “*Romanian babies all lined up.”* At the end of the Cold War in 1989, images of Romanian orphans lined up in cots caught international media attention. These children were subjected to cold, hunger, sexual abuse, physical abuse and lack of care [34].

The significant language and psychosomatic delays among these orphans later in life have been attributed to the lack of stimulation, physical contact and malnutrition [34]. Hence, it appears that much more than food is needed for healthy development.

The clinicians illustrated how children and parents are particularly affected by the body holding the trauma. The toddler bouncing off walls gradually learned to self-soothe as the mother started looking at her child’s face, particularly her eyes when she was raging. The parent’s restless legs and the labouring mother’s trauma during childbirth highlight how it is equally important for parents to work through physical trauma during the mindfulness program. Table 2 illustrates how clinicians assist parents to acknowledge and release the trauma. Parents with a trauma background often find it difficult to meditate, so the clinicians encouraged them to use mindful movement or focus their attention outside the body on an outside sound or object.

### Affect regulation mechanisms

#### Attachment

Affect regulation mechanisms included secure attachment, emotional balance, attunement, emotional awareness and emotional regulation. All clinicians emphasised the importance of attachment. Bella explicitly emphasised that reflective functioning promotes secure attachment. The others outlined how they explained attachment to the parents. Cara outlines that from the outset parents are provided information on *“What is attachment.... how it affects healthy outcomes?”* Likewise, Anna states *“We provide theory, support and a method to explore and transform attachment styles.”* A conceptual map of the attachment, abandonment, developmental needs and how “*attachment patterns are generated by your parents”* (Anna) were provided to the parents in a non-pathologizing way. “*Aversion, attachment and ignorance are predictors of mental illness”* (Bella). This perceptive observation by Bella, leads her to comment that the “*being state of mind”* promotes secure attachment. By drawing on the work of Jon Kabat-Zinn and Mark Williams, Bella articulated,

*It’s all about the being mode of mind. I mean being present and aware to your baby...that is the sort of fundamental building block to developing a secure, attuned relationship with your baby. It’s not about doing things to your babies. It’s about being with your baby.*

Thus, the “*being”* state of mind facilitates secure attachment.

Cara stated the “*facilitator provides secure attachment,” “a safe haven,”* and “*secure base”* for the parents to return each week. She uses an example of a little boy that returns each week to the teacher, even if he has not done his homework, because she does not shame or have any expectations:

*You know, think of ourselves as children, right? Eight years old and going to class, I didn’t do the assignment. But I still want to go to class. Because she loves me. You know and because I love being there.… she’ll help me and she’s not gonna shame me. Like how many of us have had that experience?*

Hence, a secure attachment with a significant attachment figure, who does not shame or reject, offers the emotional safety for children and parents to learn with confidence.

Many parents are reluctant to bring their parenting problems into the public arena. Anna stated this is a “*perception problem.”* Furthermore, parents with avoidant attachment styles are more difficult to engage. Cara described a couple where the mother was motivated, the father had an avoidant attachment style but “*both of them love* [d] *their kid.”* The mother was “*volatile with her child over nothing,”* she admitted “*I erupt… it’s really* [over] *nothing.”* The father was “*overly calm… little bit flat.”* The father would “*just sit there with his arms folded.”* The clinician provided more space and time for the father to engage. As the sessions progressed, “*When he started to open up, it got better for her* [his partner] *too.”* Thus, the reluctance some parents have with trusting the facilitator and the group is overcome by addressing their needs.

#### Emotional awareness

Increasing emotional awareness, emotional regulation and attunement were recurring themes interwoven through the four interviews. According to Anna “*emotion [is] a part of all those things”* that are group processes, mindfulness training and attachment education. However, she reiterates, “*emotion isn’t a change process. It’s the terrain of change processes…* [you] *can’t put emotion into the program.”* This comment highlights a critical point, *How do people transform?* Contrary to Anna, the other clinicians inferred emotion is a change process, that increasing emotional awareness facilitates change. Cara stated parents are encouraged to gain more awareness of their emotions by asking questions such as “*What are emotions? What is their relationship to emotions?”* Bella showed the Perinatal Anxiety and Depression Australia (PANDA) video to raise awareness about postnatal depression. Diana encouraged parents to notice the intensity and energy of depression. Self-awareness of emotions aids in gaining mastery over differentiating between different emotions, such as fear, shame, curiosity, joy and delight. Bella highlighted the temporary nature of emotions with the comment “*moods are like weather.”* Becoming aware of the temporary nature of emotions and thoughts helped parents to be less reactive.

Mindfulness offers a phenomenological methodology for parents to explore their feelings, to understand their child’s feelings and to help their child *be* with overwhelming pain (Anna). It offers parents a phenomenological exploration to experiment with feelings. “*...like MBSR, again respectful of people’s psychological defences,… putting them in the driver’s seat about how they unpack and unfold”* (Anna). Both COS and MBSR are incredibly demanding of parents to look deep inside and be the best people they can be. Placing the parents in the driver’s seat to explore themselves is empowering. Similarly, Bella reported, “*This is grist for the mill, this is all part of the process of experiential learning and knowing themselves a bit better, that sort of explorer. Being an explorer of their own subjective experiment.”* Therefore, mindfulness as a phenomenological methodology enables the user to become an explorer of emotions, to not just be with the pain, but to process it and grow from it.

#### Attunement

Three of the four clinicians also highlighted the importance of attunement in focusing on the mind of another so both “*feel felt”* and “*feel seen”* (Anna; Cara). Both Anna and Cara emphasise, “*feeling felt”* facilitates the connection between the parent and child. Bella inferred attunement through use of terms such as “*mirror neurons”* and “*reflective functioning.”* All clinicians raised issues associated with parents who have experienced trauma. Traumatised parents appear to have difficulty tuning into their child’s feeling so that the child “*feels felt”* or connected. Anna states,

*I find a lot of these parents who have had trauma don’t look at their kids in the face. Don’t actually see what is going on, so the kids feel unfelt. They feel not known, not inquired of… So I really invited him to start really catching her gaze whenever he could and just… That very important part of the COS program is delighting in the child.*

Cara describes a mother’s epiphany, “*Wow, so … I’m actually supposed to be tuning into them and filling their needs.”* When the parents start recognising the child’s needs by looking at the child’s face, a didactic shift occurs where both the parent and child start reinforcing nurturing behaviour.

#### Affect regulation

Mindfulness offers tools to assist with affect regulation, affect differentiation, containment and inhibition. Common issues beguiling parents include difficulties with state regulation, such as sleeping, settling, mis-attunement and not responding appropriately or sensitively (Bella). Anna believes mindfulness provides more support to regulate emotions than COS. Cara makes a perceptive observation that “*A child doesn’t have a strong enough nervous system to actually have self-control and they need the extended nervous system of the parent to help regulate their nervous system over and over and over again*.” Thus, the parent is the extended nervous system for the child until the child can self-regulate.

“*Emotional fireworks*” as referred to by Cara are the volatile eruptions of rage. Anna refers to this rage as the “*powerful limbic rage.*” Both Cara and Anna highlight these volatile eruptions are easily triggered in parents with traumatic backgrounds. These symptoms resemble triggers for Post-Traumatic Stress Disorder. “*It’s very hard to respond and be with the child [when you are] melting yourself”* (Cara). Containment is the ability to inhibit habitual responses, the powerful limbic rage (Anna). The aim of inhibition is affect regulation, affect differentiation, to get to know your child and not to “*blast them”* (Anna). Parents gradually learn to contain their distress by learning to *respond* rather than *react* and recognising the shark music as their underlying fears.

When parents learn emotional regulation, it models this key skill to their children. Both Diana and Bella described a case study of a four-year-old boy with autism. The mother had attended the program for her second child. When the mother used to sneak off to do meditation practice, the little boy used to follow, sit and learn the three-minute breathing space. One day, the family had been shopping and running errands. When they returned to the car, they were all “*overloaded”* and “*shaken.”* Before the father started the car, the four-year-old boy makes the sound of a meditation bell and tells the parents, “*Now I think we should all take a breathing space*. … *They actually all did the breathing space together, which was three minutes and she said it really calmed everyone down”* (Diana). This example illustrates the ease with which intergenerational transference of positive emotional regulation can occur.

#### Social learning

Social learning was another higher-order theme that emerged from the interviews. All the clinicians highlighted the usefulness of social learning and positive peer pressure. Sharing struggles, triumphs and solutions appear to promote the gaining of insight and behaviour change. The mothers “*suddenly don’t feel alone,”* they “*loved being in a group of other pregnant women”* (Diana). All the clinicians were adamant this was “*not group therapy,”* it was an adult learning class. The distinguishing feature between group therapy and adult learning appears to be that participants were not encouraged to talk at length about their concerns. The aim of the class was to teach specific skills. It facilitated vicarious learning by providing a safe, warm, supportive environment (Bella). The sharing of experiences provided group validation, which transformed their thinking. The relationship with the teacher and the group was central to practicing new behaviour (Anna). The group dynamics appear to promote respectful inquiry in a secure space (Diana). The clinicians seem to skilfully nurture the “*birth of the group”* and the ongoing group dynamics to model emotional regulation. Group processes are also relevant outside mindful parenting groups. Culture is a social learning process that influences parenting even outside of a mindful parenting group. As such, culture is a subtheme within social learning. The group dynamics appear to be akin to the “*extended nervous system,”* a connection that supports parents to alleviate their distress.

## Discussion

The aim of this study was to investigate the change processes associated with mindful parenting. The themes that emerged from the transcripts indicated reflective functioning, attachment, mind, body and social learning were important change processes associated with mindful parenting. These findings support previous research on mindfulness, parenting and phenomenology. The new theoretical model proposed by this study has the potential to expand our epistemological understanding of mindful parenting (Fig. 1). This paper focused on analysing the somatic, affective and social learning processes targeted by MP programs.

If another researcher’s analysis dramatically changed the findings, then it would be part of the theory development process. The anchor stems from a mixed method study, which synthesised findings from a systematic review, then interviewed clinicians to verify how the theory translates to practice. If the model changed after another researcher’s analysis, then it would be another credible account, not the only credible account. The final model will emerge after it has been verified by a large sample of both clinicians and parents.

The model can inform future research into the development of a more comprehensive model of mindful parenting. The anchor is simply a visual summary of change processes associated with mindful parenting. The concept can be verified by surveying a large sample of clinicians and parents through an online survey. During the initial stages of theory development, the draft model can change as the data are analysed through an iterative process. Clinicians may choose to believe the final model that has been verified by a larger sample of both parents and clinicians. Ideally, the model would be verified by biomarkers as well as psychometric measures.

This preliminary study investigated processes *associated with* mindful parenting. A Randomised Control Trial (RCT) is needed to infer processes *promoting* mindful parenting. The processes summarised in the anchor may be both processes associated with and processes causing mindful parenting. However, given the study design is not designed to infer causation, it can only suggest possible associations, from the interview data. These findings require further statistical investigation to verify association (Pearson’s correlation) and causation (RCTs).

Some MP programs have the parent and child attending the group program. Group validation is an essential part of learning to be a mindful parent as the parents learn the actual behaviours of mindful parenting in direct relation to one’s child as they observe the facilitator role modelling interactions. Behaviour is more likely to be reinforced when the group validates the behaviour and parents feel like they belong. Hence, group validation and belonging are related conceptual categories.

### Somatic mechanisms

Whole body awareness was a recurring theme in the interviews, which reinforces recent neurobiological evidence on the *embodied mind* [35]. Embodied mind refers to mindful awareness not discretely residing in the mind but residing within every cell of the body and within society [35]. All clinicians taught certain techniques to increase parents’ awareness of somatic regulation. These techniques included the body scan, the baby-body scan, “*soothing hug,”* looking at the child’s body language and looking at the child’s eyes. Terms such as the mother being the “*extended nervous system”* for the infant to regulate distressing emotions through touch, smell and voice illustrated the important role the parent plays in somatic regulation. These findings confirm the work of Bessel van der Kolk [36] and Peter Levine [37] on how trauma compromises the executive functioning (prefrontal cortex), emotional regulation (limbic system), attention regulation (thalamus) and speech (Broca’s area). The thalamus is a gatekeeper of information that has been found to be central to concentration, attention and new learning [36]. Hence, traditional talk therapies are less effective than body-based therapies, such as yoga, martial arts and singing, in releasing the physiological trauma.

According to Levine [37], traumatised individuals cannot resolve the emotional trauma until the physiological trauma has been released. This appears to be particularly relevant to the children described in this study’s interviews. A recurring theme in the interviews was the body being the “*trauma holder.”* Telling the child to control their behaviour is akin to telling embers not to explode into flames. Cooling the embers before they ignite, with a soothing voice, eye contact and providing the child with connection they yearn for were some strategies identified in the interviews. The parent being the “*extended nervous system”* for the children as they learn to regulate their emotions has not been previously reported in the literature. Tools to help the children reference their body, notice the changes in their body, particularly to find ways their body experiences power and mastery, have been found to be useful [37]. The golden route to resolving trauma is to help them experience body sensations and experiences in the body that overcome helplessness [37]. Previous research [38] indicates that “the child comes to know his body through the hands of his mother” (p. 78). The recent neurobiological evidence also shows children come to know their body through the hands and biomarkers of their mothers.

### Affect regulation mechanisms

#### Attachment

Attachment was a recurring theme in the interviews, which resonates with the contemporary parenting research. The importance of secure attachment to psychological health has been reiterated from Freud [39], Bowlby [10] to Bögels and Restifo [26]. Parental reflective functioning plays a significant role in the intergenerational transmission of attachment [40, 41]. This compassionate, nurturing interaction with the caregiver helps the child regulate own affect responses to self-soothe, allowing the child and ultimately the adult to anticipate future affect experiences without fear of being overwhelmed or rejected. Neurobiological studies now confirm the intergenerational transmission of attachment [42]. A mother’s secure attachment with her own mother has been found to promote her own increased peripheral oxytocin responses and activation of dopamine-associated reward processing brain regions, when she interacts with her infant [42]. An eloquently poignant neologism, which emerged from the interviews, portrayed the mother as an “*extended nervous system.”* This neologism has not previously been reported in the literature. It highlights the mother’s responsibility in soothing her child.

#### Emotional awareness

Mindful parenting appears to provide a phenomenological methodology for parents to understand their own and their child’s emotions without overreacting or exploding with *emotional fireworks*. Raising emotional awareness, emotional regulation and *attunement* were recurring themes for promoting positive behaviour change across the interviews, particularly the clinicians’ insights that traumatised parents had difficulties with tuning into their children’s distress and understanding their needs. Mindfulness has been found to be necessary for affective attunement between mothers and infants [43]. In fact, even before the child is born, prenatal mindfulness influenced postnatal attachment [44, 45]. Two of the clinicians also gave examples of the intergenerational transmission of emotional regulation, where a four-year-old autistic child used the three-minute breathing space to help the parents calm down.

Clinicians’ comments such as “*emotional fireworks,”* and treating the child like a “*pot plant”* illustrate traumatised mothers’ inability to read their babies’ needs. *Mis-attunement* refers to responding inappropriately to infant cues and misreading infant needs [46]. By learning to contain their own distress, parents learn to delight in their child. Siegel’s [47] use of the term *attunement* links the work of early phenomenologists, such as Husserl [48], Heidegger [49] and Satre [50], with contemporary neurobiological evidence. *Neurophenomenology* uses both mindfulness and phenomenology to examine how brain dynamics relate to conscious experience [35]. Phenomenologists explore the emotional landscape, the existential quest to understand “*being”* and to “*feel felt.”* Recent neurobiological evidence shows when one “*feels felt,”* it activates mirror neurons [51]. Hence mindfulness goes beyond attention training, it involves a fulfilling of a child’s need for connection.

Phenomenology explores existentialism as an epistemological and ontological journey to understand the nature of being, consciousness, identity and emotions. The parents learn mindfulness skills to differentiate between the “*being mode”* and the “*doing mode.”* Parents learn ‘to be’ with their child, “*to delight in their child.”* Being is the most universal yet emptiest of concepts, used by many from contemplative traditions to phenomenologists. According to Heidegger’s [49] *Being and Time*, being refers to being in the world, a state of consciousness that encompasses an underlying fundamental relationship with the world. This state is similar to Segal et al.’s [52] present-centred awareness, the *being mode* used in mindfulness practice. Heidegger’s [49] construct of ‘*being*’ also resembles the contemporary construct of the ‘embodied mind’ by Varela, Thompson and Rosch [35].

#### Social learning

Social learning was another higher-order theme illuminated by the interpretative analysis. The contribution of the group to changing the individual’s thinking was highlighted by all clinicians. These observations support research on role modelling and the importance of social context in skill development [53, 54]. Cara emphasised the mother being the “*extended nervous system”* to help the child soothe their distress. The moments of connectedness when a parent is attuned to the child make the child feel understood and accepted [55]. Likewise, the group becomes the “*extended nervous system”* to help parents regulate their own emotions.

Limitations with this study include concerns with transferability of findings and potential biases. Since only a purposive sample of four participants were interviewed, the findings cannot be generalised. Potential sources of bias could have influenced the data analysis process, even though caution was exercised to ensure the researcher’s interpretation reflected the participants’ voices. Contextual issues, such as the Skype environment, may have been blunt in capturing subtle nuances that a face-to-face interview could have captured. This study does not account for cultural differences with clinicians from other countries, which could potentially influence how individuals change in other parts of the world. Culture influences social learning within this model of mindful parenting. However, all the clinicians and their participants were mostly Anglo-Saxon Australians from educated, middle-class backgrounds. Hence, a limitation of this study was that it was unable to explore how cultural differences influence mindful parenting.

The scientific merits of this study include its rationale, conceptualisation, methodology, validity and reliability. To maintain the methodological rigor and reliability, the clinicians were given a copy of their transcripts to verify whether they agree with the content. The second author also conducted an independent audit to track the raw data to the final table. To the authors’ knowledge, the findings are reliable since the reiterative process checked that the raw data accurately reflected the researcher’s interpretation. The findings also appear to be valid as the methodology matches the research question. A qualitative research methodology is more suitable for research questions concerning “*how things are experienced”* and *“how things change”* [29]. A strength of this study is the conceptualisation of a new theoretical model of mindful parenting. With regard to a *gestalt* of mindful parenting*,* the clinicians’ insights and different change processes were synthesised into a meaningful whole to be illustrated as the anchor. The clinicians’ phenomenological accounts are consistent with the change processes identified in the systematic review. Additional change processes that emerged from the interviews include social learning.

The anchor is frequently used by mindfulness practitioners [56–58] as a metaphor ‘*to ground’, ‘to come home’* to one’s breath and body. Another shape could have been used to summarise the processes. The first stage of developing the theoretical model was to summarise the change processes identified in the systematic review. This resulted in Fig. 1, which was published in Townshend [27]. The second stage of developing the theoretical model entailed interviewing clinicians delivering MP programs. These clinicians highlighted that not all change processes were equally associated with mindful parenting. Reflective functioning was identified as having a stronger influence on developing mindfulness. Hence, the processes were organised according to the strength of their association with mindful parenting and similarity with other change processes. Therefore, the cognitive processes were grouped together, affective processes were clustered together, and body referred to somatic processes. There may be other processes that have not yet been identified. The anchor is simply a mnemonic device, a visual summary of the change processes that have been currently associated with mindful parenting.

The results of this qualitative study need to be interpreted with caution since only Australian facilitators were interviewed. The concept of secure attachment appears to be universal. However, further research is needed to clarify how cultural differences influence secure attachment and the overarching change processes embodied in the anchor. The clarification of these qualitative findings with quantitative studies has the potential to make a significant contribution to the field of mindful parenting. A longitudinal, large scale, multicentre study with vulnerable parents from diverse backgrounds that complete both psychometric assessments of change processes and physiological measures can confirm whether reflective functioning influences all other change processes, including biomarkers such as cortisol, oxytocin and dopamine [59]. It will take a significant investment to move from a small qualitative study to a large scale longitudinal multicentre study. A more reasonable step could be a single centre RCT to verify if reflective functioning influences affect regulation, attention regulation and mindful parenting. It could also clarify the impact of these change processes on the children’s developmental outcomes. Providing opportunities for research and mental health screening for all pregnant women can have far-reaching intergenerational benefits. The key clinical implication from this study is the concept of promoting reflective functioning in traumatised or vulnerable parents and policy makers.

## Conclusion

This preliminary study investigated the change processes that promote mindful parenting by interviewing four Australian clinicians of MP programs. The findings revealed six higher-order change processes, namely, reflective functioning, secure attachment, somatic regulation, social learning, cognitive processes and emotional processes. The strengths of this study include its rationale, methodology and conceptualisation of a new theoretical model. Its shortcomings include the lack of transferability and potential bias. The model is worthy of further study since it may improve the capacity to evaluate the effectiveness of MP programs. The nuanced, detailed insights from the clinicians confirmed the prevailing discourses and empirical findings on parenting, phenomenology and mindfulness. To conclude, this study conceptualised a new theoretical model embodied as the anchor to navigate the complexities of mindful parenting. For both parents and policy makers, it highlights the importance of individual and societal responsibilities in supporting parents to be the “*extended nervous system”* for their infant. The anchor has the potential to expand our understanding of how thinking, feeling and parenting can change to nurture the lost child.

## Data Availability

The de-identified datasets generated and analysed during the current study are not publicily available due to privacy policy but are available from the corresponding author on reasonable request.

## Abbreviations

CBMP: Caring For Body and Mind in Pregnancy
COS: Circle of Security
COS-M: Circle of Security and Mindfulness Based Stress Reduction
IPA: Interpretative Phenomenological Analysis
MBCT: Mindfulness Based Cognitive Therapy
MBSR: Mindfulness Based Stress Reduction
MP programs: Mindful Parenting programs
PANDA: Perinatal Anxiety and Depression Australia
